# Comparison of primary hepatic neuroendocrine tumors and non-hepatitis B non-hepatitis C hepatocellular carcinoma on contrast-enhanced ultrasound

**DOI:** 10.3389/fonc.2023.1106281

**Published:** 2023-07-10

**Authors:** Zhizhi Tan, Jiawu Li, Zhenru Wu, Zhengling Zhou, Lulu Yang, Yan Luo

**Affiliations:** ^1^ Department of Ultrasound, West China Hospital, Sichuan University, Chengdu, Sichuan, China; ^2^ Institute of Clinical Pathology, Key Laboratory of Transplant Engineering and Immunology, National Health Commission (NHC), West China Hospital, Sichuan University, Chengdu, Sichuan, China

**Keywords:** liver, primary hepatic neuroendocrine tumor, hepatocellular carcinoma, contrast-enhanced ultrasound, ultrasonography

## Abstract

**Objective:**

The purpose of this study was to compare the sonographic features of primary hepatic neuroendocrine tumors (PHNETs) to those of non-hepatitis B and non-hepatitis C hepatocellular carcinoma (NBNC-HCC) on contrast-enhanced ultrasound (CEUS).

**Materials and methods:**

Fourteen patients with a mean age of 56.9 ± 12.2 (SD) years with histopathologically confirmed PHNET were included in the study. Twenty-eight patients with a mean age of 58.5 ± 10.4 years with histopathologically confirmed NBNC-HCC were randomly selected as the control group. The clinical data, conventional ultrasound and CEUS features were retrospectively analyzed between PHNET and NBNC-HCC.

**Results:**

PHNET was more common in women (57.1%, 8/14 cases), and NBNC-HCC was more common in men (75.0%, 21/28) (*P*=0.040). No significant differences were observed in etiology, tumor marker, and liver function between the two group (*P*>0.05). Conventional ultrasound revealed that the tumor size of PHNET (10.1 ± 4.7 cm) was larger than that of NBNC-HCC (5.9 ± 3.8 cm) (*P*=0.006). NBNC-HCC was predominantly hypoechoic, while the echogenicity of PHNET varied (*P*=0.001). On CEUS, 57.1% (8/14) of PHNETs showed heterogeneous hyperenhancement, whereas 77.0% (21/28) of NBNC-HCC presented homogeneous hyperenhancement (*P*=0.015). Furthermore, 35.7% (5/14) of PHNETs showed early washout (onset of washout <60 s), which was significantly different from that of NBNC-HCC (3.7%, 1/28) (*P*=0.005).

**Conclusion:**

CEUS is helpful in discriminating between PHNET and NBNC-HCC. PHNETs mainly present as a single mass with a large size (>10 cm) in the liver. The CEUS showed that most PHNETs exhibited heterogeneous enhancement in the arterial phase, washout in the portal venous and late phases and early washout being more likely than NBNC-HCC. However, more imaging features need to be evaluated in a larger sample.

## Introduction

Neuroendocrine tumors (NETs) are a heterogeneous group of tumors that arise from neuroendocrine cells, which secrete bioactive amines and peptides. These tumors can be categorized as functional and nonfunctional. NETs are uncommon and can develop in any part of the body, with the most frequent locations being the gastrointestinal tract, pancreas, and bronchopulmonary system (1, 2). Patients diagnosed with nonfunctional neuroendocrine tumors typically lack discernible clinical symptoms, and symptoms are frequently identified only upon physical examinations. In contrast, functional neuroendocrine tumors may cause corresponding clinical symptoms due to different secreted hormones, with symptoms including hypoglycemia, diabetes, refractory peptic ulcers, abdominal pain, diarrhea, asthma and carcinoid syndrome in less than 10% of patients (3).

The liver is the most common metastatic site of neuroendocrine tumors, whereas primary hepatic neuroendocrine tumors (PHNETs) are extremely rare, accounting for 1-5% of all liver tumors and 0.8-4.0% of all neuroendocrine tumors (4, 5). Consequently, preoperative diagnoses present a formidable challenge. Imaging modalities are of significant importance in the preoperative diagnosis and postoperative follow-up of liver tumors. Magnetic resonance imaging (MRI) and computed tomography (CT) are the primary modalities utilized for abdominal diseases and can be employed to precisely locate and stage liver tumors, but the definitive diagnosis of PHNETs remains a challenge (6). Contrast-enhanced ultrasound (CEUS) has been extensively utilized for the detection and characterization of focal liver lesions owing to its real-time ability to dynamically display enhancement patterns and the degree of liver tumors. Nevertheless, only a limited number of studies have presented imaging results of PHNETs, with the majority of studies were case reports or studies with a small sample size. Moreover, there are few reports on the characteristics of conventional ultrasound and contrast-enhanced ultrasound in PHNETs (7, 8).

Previous studies have indicated that individuals with PHNET are not associated with chronic viral hepatitis, which is often misdiagnosed as liver cancer, particularly in patients lacking chronic hepatitis B and C. Furthermore, studies have shown that with the popularization of hepatitis B vaccine and the development of antiviral drugs, the prevalence of virus-related HCC is progressively declining, while the incidence of non-virus-related HCC is gradually rising (9). Hence, it is imperative to distinguish PHNETs from other liver tumors, based on distinct therapeutic alternatives and prognoses, especially concerning hepatocellular carcinoma with negative for hepatitis B surface antigen and hepatitis C antibody (NBNC-HCC). Therefore, in this study, we summarized and compared the clinical characteristics and ultrasound findings of PHNET and NBNC-HCC, with the objective of providing more imaging evidence for the diagnosis and differential diagnosis of PHNET.

## Methods

### Patient selection

This retrospective study was approved by the institutional ethics committee of West China Hospital of Sichuan University, and written informed consent was waived. Patients with histopathologically confirmed hepatic neuroendocrine tumors who underwent liver contrast-enhanced ultrasound (CEUS) in our Ultrasound Department between Mar 2011 and Jun 2022 were consecutively enrolled. The inclusion criteria were as follows: (1) hepatic neuroendocrine tumors confirmed by pathology and (2) complete clinical and ultrasound data. The exclusion criteria included clinical interventions (such as hepatic arterial chemoembolization or radiofrequency ablation) before CEUS examination; and hepatic lesions proven to be secondary hepatic neuroendocrine tumors. The clinical data, including sex, age, symptoms, serum alpha-fetoprotein (AFP), carbohydrate antigen 19-9 (CA19-9), carcinoembryonic antigen (CEA), alanine aminotransferase (ALT), total bilirubin (TBIL) and albumin levels, were collected from the hospital information system.

### Ultrasound examination

Conventional ultrasound and CEUS were performed with an iU22 ultrasound system (Royal Philips, the Netherlands) or a Resona 7 ultrasound system (Mindray Medical Solutions, Shenzhen, China) color ultrasonic diagnostic instrument equipped with a C5-1 or 5-2 MHz convex array probe, respectively. After conventional ultrasound examinations, all of the patients underwent CEUS examinations, which were performed with a real-time, low-mechanical index (0.05-0.08) imaging technique. A bolus injection of 1.2-2.4 mL of SonoVue was administered through the cubital vein, followed by flushing with 5 mL of saline. Once the injection of SonoVue was complete, the timer and video recording were started. All of the CEUS procedures were performed by physicians with at least 5 years of experience in abdominal ultrasound diagnosis. The process of CEUS contains three phases: arterial phase (0-30 s), portal venous phase (30-120 s) and late phase (>120 s).

### Imaging analysis

The conventional ultrasound images, dynamic digital video within the first minute, and typical contrast-enhanced images in the portal venous phase and late phase were subjected to independent review by two ultrasound physicians, each with at least 5 years of experience in the diagnosis of liver disease with CEUS. Discrepancies were resolved by consensus. None of the physicians were aware of the final diagnoses of the patients. In the case of patients presenting with multiple liver lesions, the most visible lesion was chosen for the analysis. The conventional ultrasound features that were assessed included the location of the lesion, its size, the number of tumors present (solitary or multiple), the echogenicity of the lesion (hypoechoic, isoechoic, hyperechoic or mixed echoic), its morphology (regular or irregular), the borders of the lesion (well-defined or ill-defined), the color Doppler signal (rare or rich) and the background of the liver (homogeneous or heterogeneous). The CEUS features that were evaluated comprised the degree of enhancement of liver lesions in comparison to the liver background at three phases (hypoenhancement, isoenhancement or hyperenhancement), the enhancement patterns of liver lesions in the arterial phase (rim-like, homogeneous or heterogeneous enhancement) and the existence of early washout (<60 seconds).

### Statistical analysis

SPSS 22.0 software (IBM, NY, USA) was used to perform the data analysis. Differences were considered significant at P<0.05. Quantitative data were expressed as the means ± standard deviations (SDs). Furthermore, categorical data were expressed as percentages. The Mann−Whitney U test was used to evaluate the differences in age distribution and tumor size between PHNET patients and NBNC-HCC patients. Categorical variables were compared with the chi-squared test or Fisher’s exact test.

## Results

**Figure 1:**
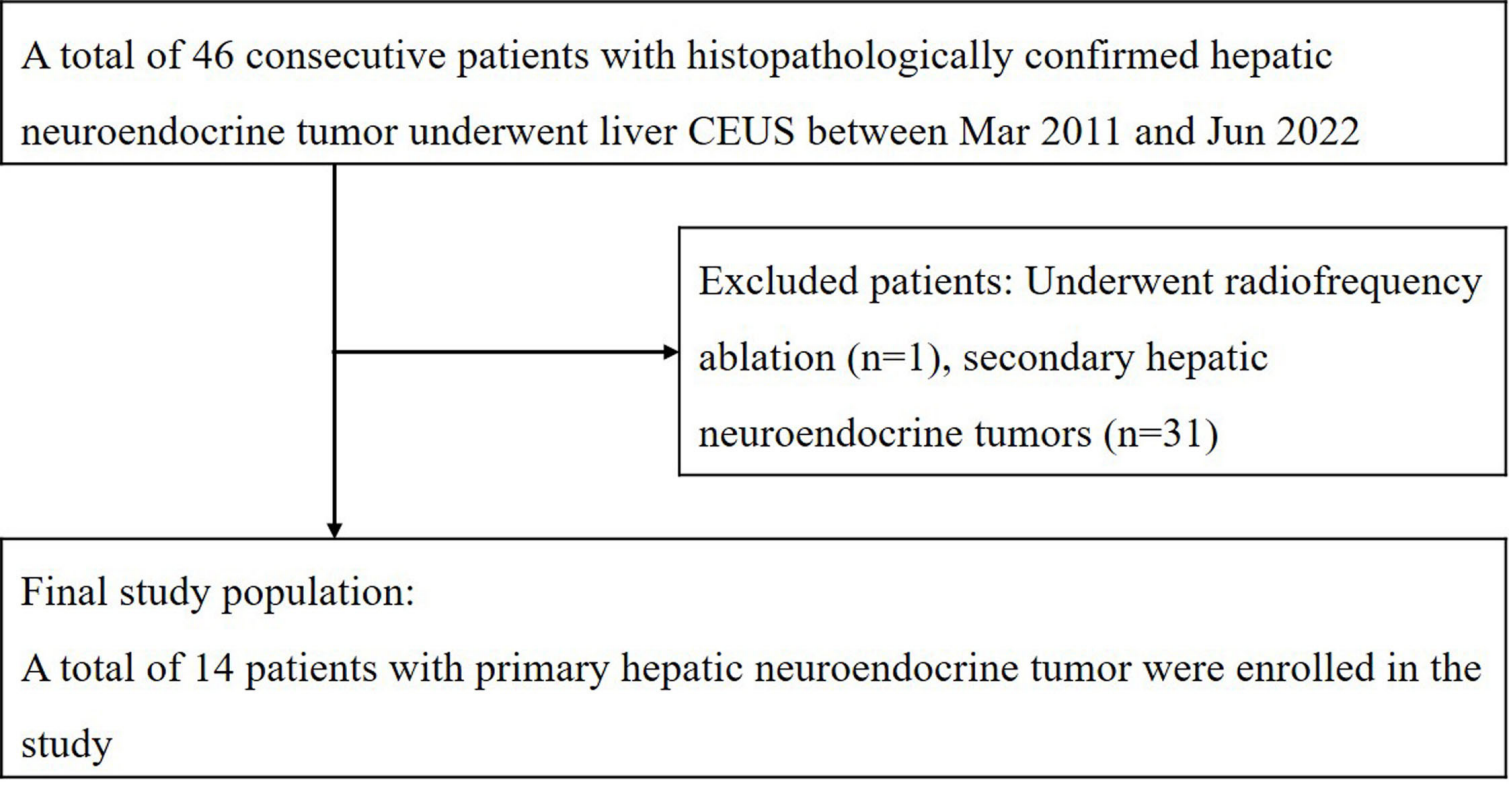
The flowchart for inclusion of patients with primary hepatic neuroendocrine tumor in the study.

During the time period, a total of 46 patients with hepatic neuroendocrine tumors confirmed by histopathology underwent liver contrast-enhanced ultrasound (CEUS) examinations. One patient underwent radiofrequency ablation before CEUS examinations, and 31 patients with a history of extrahepatic primary neuroendocrine tumors were excluded from the study. Ultimately, 14 patients with pathologically diagnosed primary hepatic neuroendocrine tumors (PHNETs) (6 men and 8 women) with a mean age of 56.9 ± 12.2 (SD) years (ranging from 32-74 years) were included in the study. **Figure 1** shows the flowchart for the inclusion of patients with primary hepatic neuroendocrine tumors in the study.

Twenty-eight patients with histopathologically confirmed NBNC-HCC (24 men and 4 women), with a mean age of 51.2 ± 10.9 (SD) years (ranging from 25-71 years) were randomly selected from our database during the same time period as the control group. All patients underwent histological confirmation through either ultrasound-guided puncture biopsy or surgical resection. A senior pathologist re-evaluated the pathological specimens for all patients to obtain definitive results (ZRW, who had at least 10 years of experience in the diagnosis of liver disease). Among them, there were two well-differentiated HCCs, 18 moderately differentiated HCCs, and eight poorly differentiated HCCs.

### Clinical and laboratory data

The tumors observed in patients with PHNET were classified according to the WHO classification as NET G1 in 2 cases (14.3%, 2/14), NET G2 in 10 cases (71.4%, 10/14) and NET G3 in 2 cases (14.3%, 2/14) (10). PHNETs were more common in women (57.1%, 8/14), and NBNC-HCC was more common in men (75.0%, 21/28) (*P*=0.004). The mean age (SD, range) of patients with PHNET (56.9 ± 12.2 years, 32-74 years) was comparable to that of patients with NBNC-HCC (58.5 ± 10.4 years, 40-78 years) (*P*=0.984). Abdominal pain was observed in 7 patients (50.0%, 7/14) with PHNET, while upper abdominal discomfort was observed in 3 patients and 4 patients were identified upon physical examination. Three patients (21.4%, 3/14) with PHNET had chronic hepatitis B. Elevated AFP serum levels were more frequently observed in patients with HCC (28.6%, 8/28), while there is no significant difference with those patients with PHNET (7.1%, 1/14) (*P*=0.111). Furthermore, there were no significant differences in the levels of carbohydrate antigen 19-9 (CA19-9), carcinoembryonic antigen (CEA), serum alanine aminotransferase (ALT), total bilirubin (TBIL) or albumin (ALB) (*P*>0.05). The clinical and laboratory characteristics of PHNET and NBNC-HCC are presented in **Table 1**.

**Table 1:** The clinical and laboratory characteristics of PHNET and NBNC-HCC in the study.

Variable	PHNET (n=14)	NBNC-HCC (n=28)	*P* value
Age (range, year)	56.9 ± 12.2 (32-74)	58.5± 10.4 (40-78)	0.984
Sex			0.040
Male	6 (42.9%)	21 (75.0%)	
Female	8 (57.1%)	7 (25.0%)	
Chronic hepatitis B	2 (14.3%)	0 (0.0%)	0.106
Chronic hepatitis C	0 (0.0%)	0 (0.0%)	—
Fatty liver disease	1 (7.1%)	6 (21.4%)	0.242
AFP (ng/ml)			0.111
≤ 20	13 (92.9%)	20 (71.4%)	
> 20	1 (7.1%)	8 (28.6%)	
CA 19-9 (U/ml)			0.350
≤ 30	11 (78.6%)	25 (89.3%)	
> 30	3 (21.4%)	3 (10.7%)	
CEA (ng/ml)			0.457
≤ 5	12 (85.7%)	26 (92.9%)	
> 5	2 (14.3%)	2 (7.1%)	
ALT (IU/L)			0.798
≤ 40	11 (78.6%)	21 (75.0%)	
> 40	3 (21.4%)	7 (25.0%)	
Total bilirubin (umol/L)			0.500
≤ 28	13 (92.9%)	24 (85.7%)	
> 28	1 (7.1%)	4 (14.3%)	
Albumin (g/L)			0.545
≤ 40	0 (0.0%)	2 (7.1%)	
> 40	14 (100.0%)	26 (92.9%)	

PHNET, primary hepatic neuroendocrine tumors. NBNC-HCC, hepatocellular carcinoma with negative for hepatitis B virus surface antigen and hepatitis C antibody; AFP, alpha-fetoprotein; CA 19-9, carbohydrate antigen 19-9; CEA, carcinoembryonic antigen. Unless otherwise stated, data are numbers of patients, with percentage in parentheses.

### Conventional ultrasound findings

The mean diameter (SD, range) of PHNETs (10.1 ± 4.7 cm, 2.0-14.4 cm) was significantly larger than that of NBNC-HCC (5.9 ± 3.8 cm, 1.5-17.0 cm) (*P*=0.006). Hypoechoic lesions were found in 6 PHNET patients (42.9%, 6/14), hyperechoic lesions were found in 4 patients (28.6%, 4/14) and mixed echoic lesions were found in 4 patients. In contrast, 89.3% (25/28) of NBNC-HCC cases were hypoechoic (*P*=0.001). A total of 64.3% (9/14) of PHNETs were located in the right liver lobe, and the other 5 cases were located in the left liver lobe. The majority of the PHNETs were solitary (85.7%, 12/14). PHNET lesions with calcifications were observed in 3 patients (21.4%, 3/14). Additionally, it was observed that the liver background of 92.9% (13/14) of PHNETs displayed homogeneous, while the remaining patient had liver damage attributable to chronic hepatitis B, which was comparable to that of patients with NBNC-HCC (*P*=0.710). However, there were no significant differences between the PHNET and NBNC-HCC groups with respect to lesion location, tumor number, morphology, tumor borders or CDFI manifestations (*P*>0.05). The comparison of conventional ultrasound features between PHNET and NBNC-HCC is presented in **Table 2**.

**Table 2:** The comparison of conventional ultrasound features between PHNET and NBNC-HCC.

Variable	PHNET (n=14)	NBNC-HCC (n=28)	*P* value
Location			0.637
Right liver	9 (64.3%)	20 (71.4%)	
Left liver	5 (35.7%)	8 (28.6%)	
Tumor size (range, cm)	10.1 ± 4.7 (2.0-14.4)	5.9 ± 3.8 (1.5-17.0)	0.006
Tumor number			0.770
Solitary	12 (85.7%)	23 (82.1%)	
Multiple	2 (14.3%)	5 (17.9%)	
Morphology			0.513
Regular	6 (42.9%)	15 (53.6%)	
Irregular	8 (57.1%)	13 (46.4%)	
Tumor borders			0.827
Well-defined	7 (50.0%)	13 (46.4%)	
Ill-defined	7 (50.0%)	15 (53.6%)	
Echogenicity			0.001
Hypoechoic	6 (42.9%)	25 (89.3%)	
Hyperechoic	4 (28.6%)	3 (10.7%)	
Mixed echoic	4 (28.6%)	0	
Lesions with calcification	3 (21.4%)	0	0.034
Color Doppler signal			0.798
rare	11 (78.6%)	21 (75.0%)	
rich	3 (21.4%)	7 (25.0%)	
Tumor in vein	0	1 (3.6%)	0.474
Liver background			0.710
Homogeneous	13 (92.9%)	25 (89.3%)	
Heterogeneous	1 (7.1%)	3(10.7%)	

PHNET, primary hepatic neuroendocrine tumors; NBNC-HCC, hepatocellular carcinoma with negative for hepatitis B virus surface antigen and hepatitis C antibody. Unless otherwise stated, data are numbers of patients, with percentage in parentheses.

### Contrast-enhanced ultrasound findings

**Figure 2:**
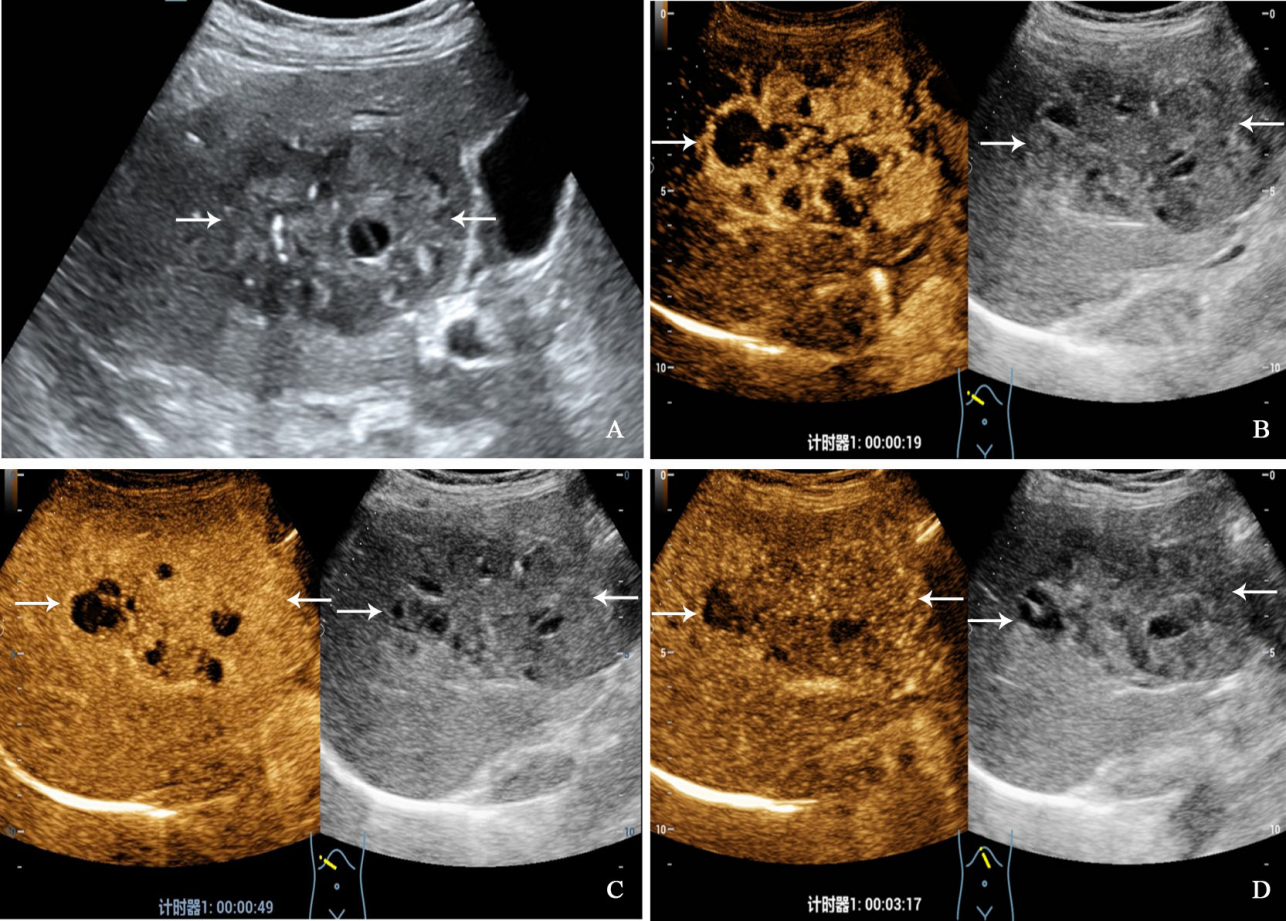
A 68-year-old woman with primary neuroendocrine tumor. The patient had no underlying liver disease. Conventional ultrasound showed that a mixed echoic tumor with focal calcification and largest diameter of 10.0 centimeters in anterior segment of the liver **(A)**. In the arterial phase of contrast-enhanced ultrasound, the tumor showed heterogeneous hyperenhancement **(B)**, and began washout before 60 seconds **(C)**. hypoenhancement in the late phase **(D)**.

**Figure 3:**
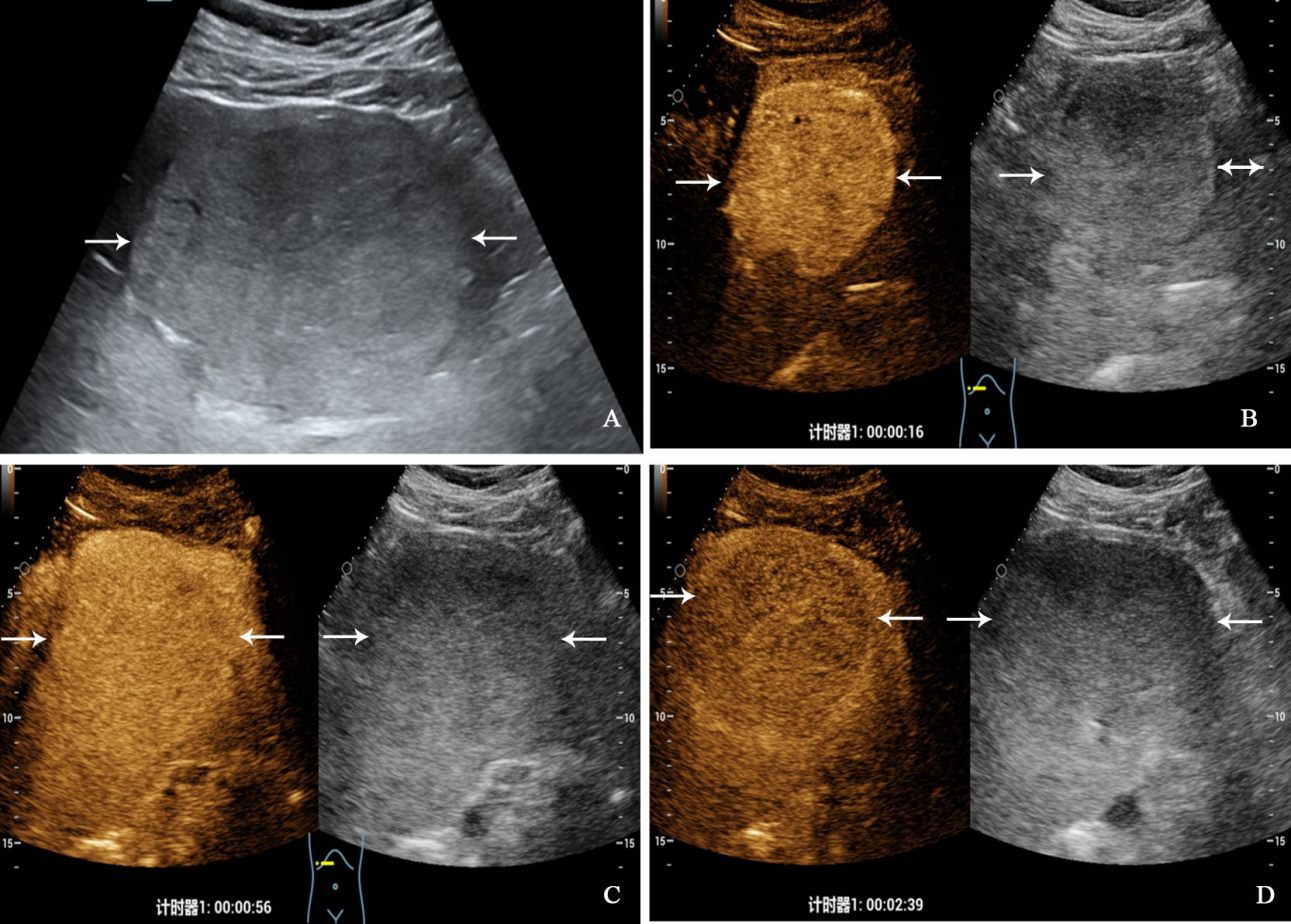
A 32-year-old man with primary neuroendocrine tumor. The patient had no underlying liver disease. Conventional ultrasound showed that a slightly hyperechoic tumor with largest diameter of 10.3 centimeters in left liver lobe **(A)**. In the arterial phase of contrast-enhanced ultrasound, the tumor showed homogeneous hyperenhancement **(B)**, and began washout at 56 seconds **(C)**, hypoenhancement in the late phase **(D)**.

**Figure 4:**
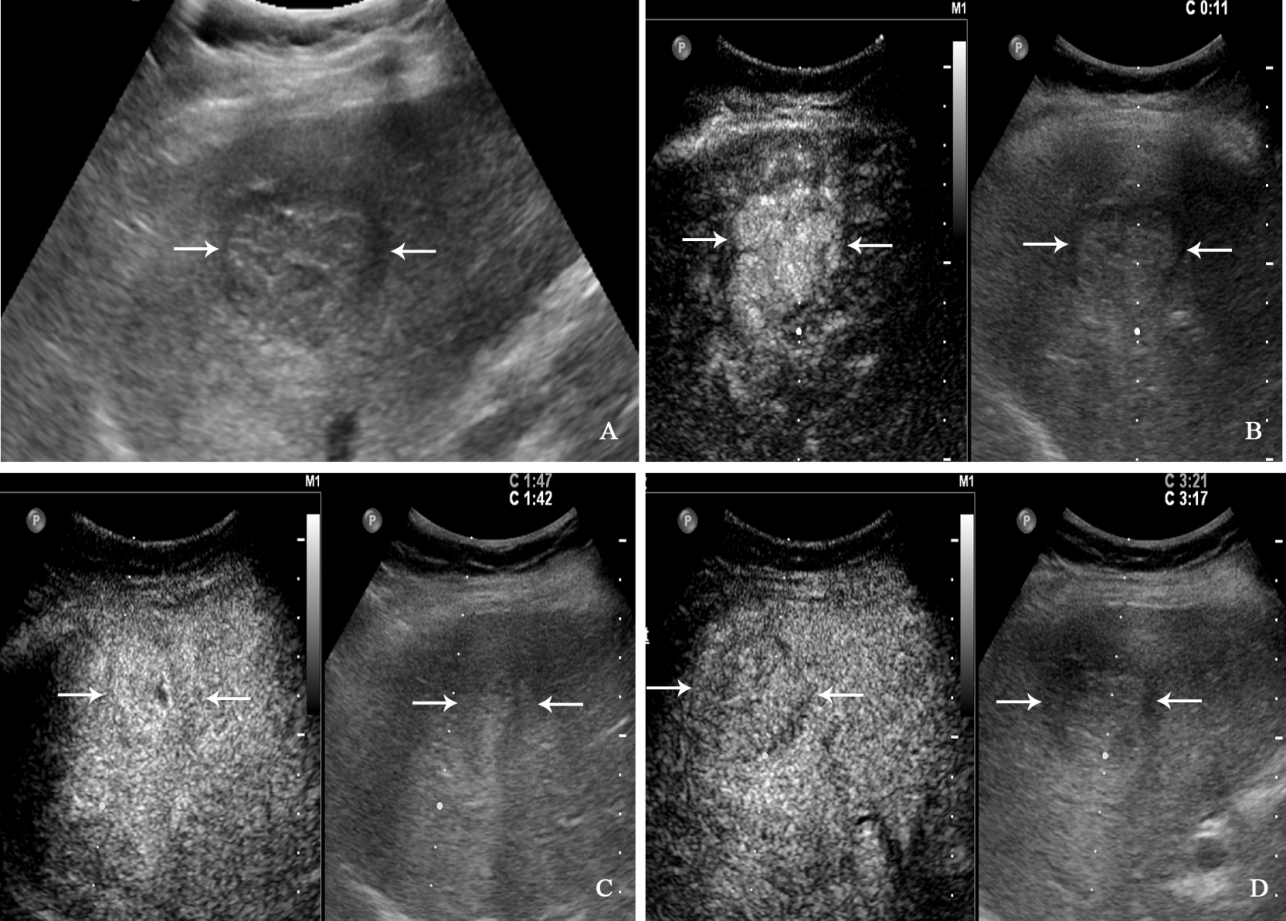
A 71-year-old man with hepatocellular carcinoma. The patient had no chronic hepatitis B and C Conventional ultrasound showed that a hypoechoic tumor with largest diameter of 3.8 centimeters in right liver lobe **(A)**. In the arterial phase of contrast-enhanced ultrasound, the tumor showed homogeneous hyperenhancement **(B)**, and isoenhancement in the portal venous phase **(C)**, finally with slightly washout in the late phase **(D)**.

All patients underwent CEUS examinations. On CEUS, both PHNET and NBNC-HCC exhibited hyperenhancement (100%) in the arterial phase. In terms of enhancement patterns in the arterial phase, 57.1% (8/14 cases) of PHNETs showed heterogeneous enhancement (**Figure 2**), while 2 cases (2/14, 14.3%) exhibited rim-like enhancement and 4 cases (4/14, 28.6%) exhibited homogeneous enhancement (**Figure 3**). However, 75.0% (21/28) of NBNC-HCC exhibited homogeneous enhancement (**Figure 4**), and 21.4% (6/28) exhibited heterogeneous enhancement in the arterial phase, which is significantly different from PHNET (*P*=0.015). There was no significant difference in the enhancement degree of CEUS in the portal phase and delayed phase (*P*>0.05). However, 35.7% (5/14) of PHNETs demonstrated early washout (onset of washout <60 s), which was significantly different from that of NBNC-HCC (1/28, 3.7%) (*P*=0.005). The comparison of contrast-enhanced ultrasound characteristics between PHNET and NBNC-HCC is presented in **Table 3**.

**Table 3:** The comparison of contrast-enhanced ultrasound characteristics between PHNET and NBNC-HCC.

Variable	PHNET (n=14)	NBNC-HCC (n=28)	*P* value
Enhancement pattern in arterial phase			0.015
Rim-like enhancement	2 (14.3%)	1 (3.6%)	
Homogeneous enhancement	4 (28.6%)	21 (75.0%)	
Heterogeneous enhancement	8 (57.1%)	6 (21.4%)	
Arterial phase			—
hyperenhancement	14 (100.0%)	28 (100.0%)	
iso- or hypoenhancement	0	0	
Portal venous phase			0.172
hyperenhancement	0	2 (7.1%)	
isoenhancement	3 (21.4%)	10 (35.7%)	
hypoenhancement	11 (78.6%)	16 (57.1%)	
Early washout (<60 sec)	5 (35.7%)	1 (3.7%)	0.005
Late phase			
Isoenhancement	0	1 (3.7%)	0.474
hypoenhancement	14	27 (96.3%)	

PHNET, primary hepatic neuroendocrine tumors; NBNC-HCC, hepatocellular carcinoma with negative for hepatitis B virus surface antigen and hepatitis C antibody. Data are numbers of patients, with percentage in parentheses.

## Discussion

Primary hepatic neuroendocrine tumors (PHNETs) are extremely rare in clinical practice, yet their incidence has risen in recent years (1, 2). PHNETs are a type of tumor with abundant blood supply and are easily confused with hepatocellular carcinoma (3, 11). In this study, we retrospectively analyzed the clinical manifestations and conventional ultrasound and CEUS characteristics of PHNET and NBNC-HCC, and the results showed that there were some differences between PHNET and NBNC-HCC in clinical and ultrasound characteristics.

The data from the Surveillance, Epidemiology, and End Results (SEER) database indicates that PHNET is more prevalent in women (54%), with a median age of 63 years (12), and 57.1% of patients were female in our study, with a mean age of 56.9 years. Due to the fact that PHNET patients often present without specific symptoms, resulting in larger tumor size at clinical detection (13–15). The average diameter of PHNETs in the study was 10.1 ± 4.7 cm, which is significantly larger than that of NBNC-HCC. Furthermore, the tumor markers of PHNET patients did not exhibit any distinctive alterations, and in the majority of patients, the tumor markers (namely AFP, CA19-9 and CEA) remained within the normal reference range (7). In this study, only one patient who had chronic hepatitis B exhibited a slight increase in AFP, which could be attributed to inflammatory changes in the liver. Additionally, 21.4% and 14.3% of patients in this study had elevated CA19-9 and CEA, respectively, which is in accordance with previous studies (7, 16). However, our study showed there were no statistically significant differences in terms of etiology, tumor markers, and liver function between PHNET and NBNC-HCC groups, indicating that it is difficult to distinguish the two groups based on laboratory tests alone.

Currently, there is limited research available regarding the ultrasound manifestations of primary hepatic neuroendocrine tumors, and the majority of existing studies being comprised of case reports (7, 8, 17). Our study found that 85.7% of PHNETs were solitary, which is consistent with previous studies (5, 13, 18). The echogenicity of PHNETs was found to be variable, with 42.9% being hypoechoic, 28.6% hyperechoic and 28.6% mixed echoic, which is significantly different from NBNC-HCC (89.3% were hypoechoic). Li et al. (7) reported that 60% of PHNETs were hyperechoic, whereas 30% demonstrated mixed echogenicity in a cohort of ten patients. In contrast, a different investigation revealed that 83.3% of PHNETs displayed mixed echogenicity, and suggested that cystic changes frequently occur in PHNETs (8). Therefore, cystic change may represent a feature of PHNETs (19), although this requires confirmation in a larger cohort. Occasionally, calcifications (21.4%) were also present in PHNETs, which is different from NBNC-HCC. However, our study showed that there were no significant differences between PHNET and NBNC-HCC in terms of lesion location, tumor number, morphology, tumor borders and CDFI manifestations.

On CEUS, although PHNET and NBNC-HCC both presented hyperenhancement in the arterial phase, there were some differences in the enhancement patterns between these tumor types. In this study, 57.1% of PHNETs demonstrated heterogeneous enhancement, 14.3% exhibited rim-like enhancement and 28.6% exhibited homogeneous enhancement. However, 75.0% of NBNC-HCC was homogeneously enhanced, while 21.4% was heterogeneously enhanced in the arterial phase. This discrepancy may be attributed to the higher prevalence of cystic changes in PHNET compared to NBNC-HCC. Furthermore, our study revealed that 14.3% of PHNETs exhibited rim-like enhancement in the arterial phase, which was different from the study of Chen et al. (6) where 66% (6/9 cases) of PHNETs displayed peripheral enhancement. The cause for this discrepancy is uncertain, and it could potentially be linked to the tumor grade. In their study, all PHNETs were graded as G3, whereas only 2 cases were graded as G3 in our study. Further studies are needed to determine if there are variations in the enhancement patterns of PHNETs with different grades. Moreover, all of the PHNETs were observed to be washed out in either the portal venous or late phase, which is a typical feature of malignant liver lesions. This renders it challenging to distinguish PHNETs from NBNC-HCC. However, 35.7% of PHNETs showed an early washout in the portal venous phase, which was significantly different from NBNC-HCC (3.7%) in our study. The Contrast-Enhanced Ultrasound Liver Imaging Reporting and Data System (CEUS LI-RADS) algorithm, established by the American College of Radiology (ACR), has been utilized to assess the possibility of HCC in focal liver lesions. These PHNETs with early washout will be classified as LR-M (probably or definitely malignant, not necessarily HCC) by CEUS LI-RADS algorithm, which differs from the classification of HCC as LR-5. The CEUS LI-RADS algorithm can serve as an auxiliary tool for lesion nature assessment during practical clinical work. Additionally, Li et al. (7) reported that 80% of PHNETs exhibited rapid washout, with a median onset washout time of 81 seconds. It is well established that the majority of HCCs arise in individuals with cirrhosis, which may be induced by hepatitis B or C virus or alcohol abuse (20, 21). However, 21.4% of PHNET patients had chronic hepatitis B in our study, and the differential diagnosis of PHNET and hepatitis virus related HCC remains challenging in these patients.

Each imaging modality is not isolated. In clinical practice, special attention is required when diagnosing HCC in high-risk patients with a single liver lesion, as HCC is the only solid tumor that can be identified through contrast-enhanced imaging. Therefore, it is essential to incorporate the patient’s clinical or epidemiological history, tumor markers, and other relevant factors to prevent misdiagnosis of non-malignant tumors as HCC (22). Meanwhile, in cases where diagnosis proves challenging, biopsy remains necessary to confirm the nature of the lesion. Complementary to its ability to delineate enhancement patterns of PHNET, CEUS can effectively discern active and necrotic regions of tumors, thereby guiding biopsy procedures and facilitating the acquisition of an optimal biopsy sample (23). The pathological results obtained through biopsy remain the preferred method for determining focal liver lesions. This approach not only enables identification of the nature of the lesions, but also allows for accurate grading of the neuroendocrine tumors (NETs) (24). Consequently, such results serve as crucial evidence for guiding further clinical management.

There were several limitations in our study. Firstly, this was a retrospective study conducted at a single center, and the number of cases were relatively small due to the low incidence of PHNETs. Secondly, the presence of selection bias was unavoidable as the control group was not matched perfectly. Thirdly, due to the limited number of cases, further study of the ultrasound features comparison between different differentiation degrees of HCC and PHNET was not conducted. Finally, secondary hepatic neuroendocrine tumors were not included in this study, and further study is necessary to explore potential differences between primary and secondary neuroendocrine tumors.

In conclusion, our study showed that CEUS is helpful in discriminating between PHNET and NBNC-HCC. PHNETs mainly present as a single mass with a large size (>10 cm) in the liver. CEUS showed that most PHNETs showed heterogeneous enhancement in the arterial phase, washout in the portal venous and late phases and early washout being more likely than NBNC-HCC. However, more imaging features need to be studied in a larger sample.

## Data availability statement

The original contributions presented in the study are included in the article/supplementary material. Further inquiries can be directed to the corresponding author.

## Ethics statement

The studies involving human participants were reviewed and approved by Institutional Ethics Committee of West China Hospital of Sichuan University. Written informed consent for participation was not required for this study in accordance with the national legislation and the institutional requirements.

## Author contributions

ZT analyzed the date and wrote the paper. ZW, JL and ZZ track the paper. ZT, ZW, JL, ZZ, LY and YL collected the data. All authors read and approved the final manuscript.
